# DEVELOPING A MODEL FOR COLLEGE STUDENT VOLUNTEERING IN RESPITE CARE

**DOI:** 10.1093/geroni/igae098.3027

**Published:** 2024-12-31

**Authors:** Marta Rodriguez-Galan

**Affiliations:** St. John Fisher University, Rochester, New York, United States

## Abstract

The purpose of our study is to evaluate college student volunteer’s learning on aging and caregiving through EXHALE, a grant-funded pilot respite program for family caregivers developed by Lifespan or Greater Rochester and St. John Fisher University. We are sharing the results from the first year of student participation (the program will be funded for 3 years). In addition to promoting student service learning in the field of aging, this evaluation will help in the development of a toolkit that can be used by other higher education institutions. The specific aims of the evaluation research are: 1) To evaluate students’ changes regarding attitudes towards aging, specifically towards frail older adults 2) To evaluate specific areas of learning regarding aging that were more salient in their volunteer experience (e.g. working with older adults with dementia, working with low-income minority elders, etc.) 3) To identify content areas for the development of the “toolkit” that will be used as a model for training future volunteers in higher education institutions. To assess student learning in the program we used the following three

**methods:**

1) The Kogan’s attitudes towards older people scale; 2) Student Reflections after each volunteer session at the respite care; 3) Bi-weekly debrief sessions with the professor. Furthermore, one of the student volunteers also served as a research assistant during the summer and received more personalized mentoring. Our findings show that students challenge some of their initial attitudes on aging, and gain knowledge about caregiving and cultural competency through this volunteering program.

